# Screwworms, *Cochliomyia hominivorax,* Reared for Mass Release Do Not Carry and Spread Foot-and-Mouth Disease Virus and Classical Swine Fever Virus

**DOI:** 10.1673/031.008.6201

**Published:** 2008-10-23

**Authors:** M. F. Chaudhury, G. B. Ward, S. R. Skoda, M.Y. Deng, J. B. Welch, T. S. McKenna

**Affiliations:** ^1^ USDA-ARS, Screwworm Research Unit, Panama City, Panama; ^2^ USDA-APHIS, Veterinary Services, FADDL, Plum Island Animal Disease Center, Plum Island, New York; ^3^ USDA-ARS, KBUSLIRL, Kerrville, Texas

**Keywords:** New World screwworm, formaldehyde, rearing, strain development, viral disease transmission

## Abstract

Experiments were done to determine if transporting live screwworms *Cochliomyia hominivorax* Coquerel (Diptera: Calliphoridae) for developing new strains from countries where foot-and-mouth disease and classical swine fever are endemic, to the mass rearing facilities in Mexico and Panama, may introduce these exotic diseases into these countries. Are screwworms capable of harboring and spreading foot-and-mouth disease virus (FMDV) and classical swine fever virus (CSFV) when they are grown in virus-inoculated larval rearing medium? In one experiment, screwworm larvae were reared in a FMDV-inoculated artificial medium containing either 0.1 % formaldehyde or antibiotics as an antimicrobial agent. In another experiment, larvae were similarly reared in a CSFV-inoculated artificial medium containing 0.1% formaldehyde. In each experiment, samples of larvae and the rearing media were collected daily until pupation occurred. The presence of FMDV was assayed by observing cytopathic effects on cell cultures and a conventional reverse transcription-polymerase chain reaction (RT-PCR); CSFV was assayed using an avidin-biotin complex assay and a conventional RT-PCR. For media containing antibiotics, FMDV was detected in a larval sample collected on day 1 and in media samples on days 1, 2 and 3. No FMDV was detected from larval and media samples collected on all other days. For media containing formaldehyde, FMDV and CSFV were not detectable in larval or media samples collected on all sampling days. These results indicate that FMDV and CSFV cannot survive in rearing medium containing formaldehyde as an antimicrobial agent. Therefore, insects collected in endemic regions and reared using formaldehyde-containing medium for at least one generation at the collection site should be free of FMDV and CSFV and can be transported safely to a strain development/mass rearing facility.

## Introduction

Screwworms, *Cochliomyia hominivorax* Coquerel (Diptera: Calliphoridae), cause myiasis in cattle, wildlife and humans. This insect has been eradicated from the United States, Mexico and most of the Central America, using sterile insect technology. Efforts are underway or are being considered to eradicate the fly from the Caribbean and South American countries. The continued success of the eradication program requires the development of new screwworm strains from insects collected from these countries. Highly contagious viral diseases of livestock, such as foot-and-mouth disease and classical swine fever are endemic in these areas. Foot-and-mouth disease virus (FMDV) and classical swine fever virus (CSFV) are the causative agents for these diseases, respectively. Both of the viruses are known to spread through contact (Katz et al. 1993; Murphy et al. 1999). However, it is not known whether screwworms or any other insect, if and when they come in contact with these viruses, can carry them and serve as a potential vector for transmitting these diseases. It is important, therefore, to determine whether transporting live screwworm material to develop new strains from a disease endemic area to the screwworm mass rearing facilities in Mexico and Panama could introduce an exotic disease of livestock to these countries. Research on insects and other ectoparasites, and their vectoral capacity to transmit these pathogenic diseases through mechanical or trans-ovarial transmission mode, is of importance in relation to implications of such organisms as potential vectors in acts of bioterrorism.

Therefore, the purpose of present study was to investigate if screwworms are capable of harboring FMDV and CSFV when they are grown in virus-inoculated rearing media and to evaluate strategies for eliminating the likelihood of introduction of exotic disease agents through the movement of screwworm materials from endemic countries.

## Materials and Methods

### Rearing of insects and sampling

Screwworm larvae (strain Panama-95) were reared using standard rearing procedures (Chaudhury and Alvarez 1999). For experiments with FMDV, newly hatched larvae were reared to maturity in a diet containing 0.1% formaldehyde (0.1ml of 37% formaldehyde per 100 ml of medium), and in a diet containing antibiotics and fungicide (200 units of penicillin, 200 ***µ*** g of streptomycin, 0.1 mg of gentamicine and 0.25 ***µ*** g of fungizone amphotericin B per ml of medium), respectively. For 100 ml of each type of rearing media, 2 ml of FMDV, serotype O1, strain South Korea, were added for a final concentration of 106 TCID50 (50% tissue culture infective dose) per ml of the medium. For experiments with CSFV, newly hatched larvae were reared to maturity in a diet containing 0.1% formaldehyde only and the virus was added to the medium for the same concentration as for the FMDV. The rearing media were held in round 10 × 13 cm plastic containers. For all experiments, approximately 300 newly hatched screwworm larvae were introduced into 200 ml of each of the rearing media prepared as above. Controls were set up for each medium with screwworm larvae but without the viruses. Controls were also set up for each medium with FMDV or CSFV but without screwworm larvae.

On the day of the experiment and everyday thereafter, 2 samples of 20 larvae each were collected from each test diet. Larvae of one of the samples were rinsed with distilled water only, and those of the other sample were rinsed with distilled water, 10% acetic acid and 70% ethanol in sequence for a minute in each liquid. At the same time, a sample of diet medium from each test was taken. All samples were collected in 1.5 ml Eppendorf tubes, sealed, labeled and stored in -70°C until processed. After taking samples, 100 ml of fresh diet was added each day to each of the containers with the remaining screwworm larvae resulting in a total of 500 ml diet per container until larval maturation. The pH of the medium in each container was recorded daily during the sampling period using an Extech waterproof digital pH tester for semi liquid-semisolid substances (Daigger Laboratory Equipment Supplies, www.daigger.com). These tests were repeated two times.

### Virus extraction from larvae

About 1g of larvae were homogenized in a Mixer Mill (Retsch Type MM300, Retsch, Inc., www.retsch.com) at a concentration of 200 mg larvae/ml of BioWhittacker™ Eagle's Minimum Essential Medium (EMEM, Cambrex BioScience, www.cambrexcom). Particulate materials were removed from the homogenate by centrifugation and the supernatant was collected. The supernatant was filtered through a 0.45 ***µ***m Spin-X® Centrifuge Tube Filter (Corning Incorporated, www.corning.com/lifesciences). BHK-21 and LK cells were used for FMDV isolation and SK-6 cells were used for CSFV isolation. Cell cultures were inoculated with a volume of 100 ***µ*** l of the filtered larval extract for virus isolation.

### Virus extraction from media

About 2 g of the medium were centrifuged at 2,000 rpm for 30 min in a Model J6B centrifuge (Beckman Instruments, Inc., www.beckman.com). The supernatant was transferred to Eppendorf tubes and centrifuged in a microfuge at 14,000 rpm for 10–15 min. The resulting supernatant was then transferred to a 0.45 ***µ*** m Spin-X® Centrifuge Tube Filter and centrifuged at the maximum speed for 10 min. The filtrate was then used for virus isolation on cell cultures as described above.

### Detection of FMDV

FMDV was detected by observing cytopathic effect on cell cultures for two passages. Test results in cell cultures were confirmed by a conventional reverse transcription-polymerase chain reaction (RT-PCR) and an antigen enzyme-linked immunosorbent assay (ELISA). For the conventional RT-PCR, FMDV RNA was extracted with the TRIzol® LS Reagent (Invitrogen Corporation, www.invitrogen.com) following the manufacturer's instructions. The RT-step of the RT-PCR for FMDV was the same as described by Deng et al. (2005). The PCR step of the RT-PCR for FMDV was carried out in a volume of 25 ***µ*** l including 5 ***µ*** l of RT product, 2 ***µ*** l of 10X buffer, 300 nM each of the two primers and 0.625 units of Ampil*Taq*® DNA polymerase. The sequence of the forward PCR primer for FMDV was AACCAGATGCAGGAGGACAT, whereas the sequence of the reverse PCR primer for FMDV was TTGTACCAGGGTTTGGCTTC. The reaction mixtures were subjected to 31 cycles at 95°C for 30 seconds, 55°C for 30 seconds and 72°C for 30 seconds, with an additional 3-minute extension at 72°C. A 10-***µ*** l portion of RT-PCR products was analyzed by agarose electrophoresis with ethidium bromide staining. A sample was considered positive for FMDV when it generated PCR products of the expected size of 131 bp in the conventional RT-PCR for FMDV. The antigen ELISA was conducted as described in the Manual of Diagnostic Tests and Vaccines for Terrestrial Animals (mammals, birds and bees) of World Organization of Animal Health (2004).

### Detection of CSFV

CSFV was detected by an avidin-biotin complex (ABC) assay and a conventional RT-PCR. For the ABC assay, CSFV-infected SK-6 cells on slides were fixed with a 60:40 mixture of acetone: methanol for 10 minutes at 20°C. A 1% normal horse serum was added to the slides and the slides were incubated on a level surface in a humidified chamber at 37°C for 5 minutes. The normal horse serum was shaken off from the slides and a 1:1,000 dilution of CSFV specific monoclonal antibodies VP-3 (CID-DLO, Lelystad, The Netherlands) was added. The slides were incubated in the humidified chamber at 37°C for 30 minutes. The slides were rinsed in 0.01 M phosphate buffered saline with 0.05% Tween 20 (PBST), pH 7.2 for three times. A working solution of biotinylated horse anti-mouse antibody was added to the slides and the slides were incubated in the humidified chamber at 37°C for 30 minutes. The slides were washed three times as above. An ABC reagent was prepared according to the manufacturer's instructions of the Vectastain ABC Alkaline Phosphatase Kit for Mouse IgG (Vector Laboratories, www.vectorlabs.com) and added to the slides. The slides were incubated at 37°C for 30 minutes and then washed twice in PBST. A substrate indicator was prepared in Tris-HCl, pH 8.2 and added to the slides according to manufacturer's instructions (Vector Red Alkaline Phosphatase Substrate Kit, Vector Laboratories). The slides were incubated at room temperature or 37°C for 20–30 minutes in the humidified chamber. The slides were rinsed in distilled water for 1 minute and then counterstained with Gill's #2 haematoxylin for 1 minute. The slides were then rinsed twice in distilled water and mounted with a cover slip and Vectamount mounting medium. The slides were read under a microscope. The RT-PCR for CSFV was conducted as described by Deng et al (2005).

## Results

No FMDV or CSFV were detected in any of the samples examined from all the tests except in one larval sample and a media sample in one of the tests as shown in Table 1. In the experiment on FMDV with the rearing medium containing antibiotics, FMDV was detected only in a larval sample collected on day 1 and rinsed with only distilled water (Table 1); the virus was also detected in the same media samples on days 1, 2 and 3 by virus isolation. No FMDV was detected in larval samples collected on all sampling days that had been washed with distilled water, acetic acid and ethanol in sequence. Results of virus isolation in cell cultures were confirmed by the conventional RT-PCR for FMDV.

In the experiment on FMDV with the rearing medium containing formaldehyde, no FMDV was detected in larvae reared in the medium with formaldehyde whether they were washed with just distilled water or with distilled water, acetic acid and ethanol in sequence. Similarly, no FMDV was detected in any corresponding media samples (Table 1).

In the experiment on CSFV with the rearing medium containing formaldehyde, virus detection was carried out only on the larval samples reared in formaldehyde containing media and were washed with distilled water, acetic acid and ethanol in sequence. No CSFV was detected in any of the larval samples (days 1–5) or in the corresponding media samples except that the medium sample collected on day 1 was positive in the RT-PCR for CSFV (Table 1 This sample was negative for the virus in the ABC assay for CSFV indicating that the virus in the sample had been inactivated in the medium.

## Discussion

No similar study has been conducted to investigate vector potential of screwworms or other livestock insects for carrying and/or transmitting foot-and-mouth disease or classical swine fever viruses except one mention of the presence of FMDV in insects in Russia (Poliakov and Rozov 1970). Development and persistence of pathogenic bacteria such as *Escherichia coli* in flies such as the housefly, *Musca domestica,* stable fly, *Stomoxys calcitrans,* and other muscoid flies has been studied more extensively (Greenberg 1959, 1962; Rochon et al. 2004, 2005). These studies clearly indicate that *E. coli* persists throughout metamorphosis into the adult flies where it could more readily be disseminated throughout the environment (Rochon et al 2005). Prion diseases, such as chronic wasting disease and scrapie (in sheep), are neurogenerative diseases of humans and animals. Chronic wasting disease is endemic and has affected thousands of domestic and wild cervids in the US. The mode of transmission is not known, although direct contact between infected and non-infected animals via saliva, urine and feces has been considered. Because house fly larvae exposed to infected brain material were able to readily transmit scrapie to hamsters, it has been suspected that flies and other ectoparasites may play a role in transmission of these diseases (Lupi 2005). Results reported from the present study help to develop a better picture of possible roles of insects and other ectoparasites in transmitting pathogenic diseases of cattle and humans.

**Table 1.**
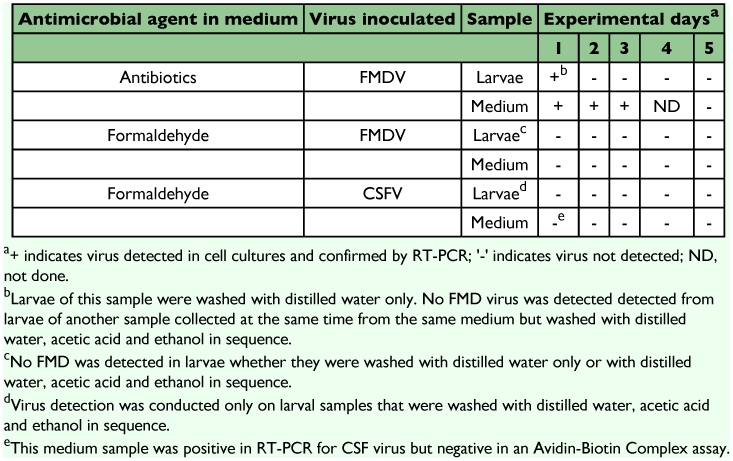
Virus detection in screwworm and media samples

In Screwworm rearing facilities in Mexico and Panama, the formaldehyde-containing medium is routinely used. The main purpose of our experiments was to learn if screwworms can carry these FMDV and CSFV when they are grown in this medium inoculated with these viruses. Therefore, experiments were conducted on both FMDV and CSFV in formaldehyde-containing medium. The inclusion of experiments on FMDV in antibiotics-containing medium was of less significance to the goal of the study. No experiments were conducted on CSFV with media containing antibiotics.

FMDV was temporarily stable in rearing medium containing antibiotics, but insects reared in medium with formaldehyde were free of FMDV. In medium containing antibiotics and FMDV, the virus was detected in a larval sample collected on day 1 and washed with distilled water only and no virus was detected in larval samples collected on all subsequent sampling days and washed with distilled water, acetic acid and ethanol in sequence. This suggests that the virus can be carried on the outside of larvae reared in the medium since viable virus was not present when the outside of the larvae was surface sterilized

Survival of CSFV was studied in rearing medium containing formaldehyde and not with antibiotics. This was because the preliminary experiments and the experiments with FMDV suggested that the larvae reared in formaldehyde-containing media would provide us with virus-free samples. CSFV was not detectable by virus isolation from formaldehyde-containing medium inoculated with the virus, or from insects reared in the medium. Genomic RNA was detected in one of the medium samples collected on day 1 of the experiment. However, a positive PCR does not necessarily require the presence of a live virus. Since CSFV is an enveloped virus, the RNA genome may be briefly protected from degradation in the medium.

These results clearly indicate that the FMDV and CSFV cannot survive in the rearing medium that contains formaldehyde as an antimicrobial agent. Formaldehyde is widely used as the antimicrobial agent in rearing media of the screwworm program to prevent microbial growth, particularly fungal contamination of the media. Therefore, insects collected in endemic regions and reared in the formaldehyde-containing medium for at least one generation at the collection site should be free of FMDV and CSFV, allowing safe transport of newly collected screwworms as pupae to the strain development/mass rearing facility. According to Favero and Bond (2001), formaldehyde is often used as an antimicrobial agent against viruses, such as FMDV and CSFV. It has a broad-spectrum of action, and its mode of action is by alkylation with amino and sulfhydryl groups of proteins and ring nitrogen atoms of purine bases.

In conclusion, these results indicate that screwworms, when they come in contact with animals infected with FMDV or CSFV, may become infected and be potential carriers of these viruses. To prevent the spreading of disease, particularly during collection of screwworm samples for developing new strains for mass rearing, care must be taken so that the potential carrier organisms must go through a decontamination process, such as rearing screwworms in formaldehyde-containing medium, before transporting them to disease free areas or countries.

## Abbreviations

ABC: avidin-biotin complex
CSFV: classical swine fever virus
FMDV: foot-and-mouth disease virus
RT-PCR: reverse transcription-polymerase chain reaction
